# Activated Carbon Functionalization for Lipase Immobilization: Characterization, Hydrolytic Activity, and Ethyl Lactate Synthesis

**DOI:** 10.1007/s12010-026-05810-x

**Published:** 2026-07-01

**Authors:** Priscilla Amaral Nascimento, Jéssica Ferreira Borges, Mateus Pereira Flores Santos, Annie Nolasco Alves, Leandro Soares Santos, Rafael da Costa Ilhéu Fontan, Renata Cristina Ferreira Bonomo, Wenrong Yang, Cristiane Martins Veloso

**Affiliations:** 1 Process Engineering Laboratory, State University of Southwestern Bahia, Itapetinga, 45700-000 Bahia Brazil; 2 Department of Natural Science, State University of Southwestern Bahia, Vitoria da Conquista, 45083-900 Bahia Brazil; 3 Department of Animal and Rural Technology, State University of Southwestern Bahia, Itapetinga, 45700-000 Bahia Brazil; 4 School of Life and Environmental Sciences, Deakin University, Geelong, VIC 3216 Australia; 5 Department of Agroindustry, Federal University of Sergipe, Nossa Senhora da Gloria, Sergipe, 49680-000 Brazil

**Keywords:** Aroma ester, Functionalization, Covalent bond, Genipin, Metallic particles

## Abstract

This study aimed to evaluate the effect of different surface modifications of activated carbon: genipin (GAC), iminodiacetic acid associated with metal particles (MAC), and the combination of genipin with metal particles (GMAC). For the immobilization of two lipases (porcine pancreatic lipase – PPL and *Candida rugosa* lipase – CRL). In addition, the applicability of the resulting biocatalysts was investigated using ethyl lactate synthesis as a model esterification reaction (40 °C/4 h, with a 1:3 molar ratio of lactic acid to ethanol). All adsorbents showed immobilization yield higher than 87%, with metalized activated carbons exhibiting the highest hydrolytic activities for both immobilized enzymes (48.1 U for PPL and 51.6 U for CRL). The immobilized derivatives achieved ethyl lactate conversions above 90% for both enzymes, with PPL GMAC reaching 94.2%, comparable to the native enzyme (94.9%). Moreover, the derivatives-maintained ester conversions above 80% after five consecutive reaction cycles under organic reaction conditions (GMAC 85.8% for PPL; GMAC 88.1% for CRL), indicating that the immobilization strategy effectively reduced enzyme desorption while preserving catalytic activity. Therefore, the synthesized derivatives demonstrated potential as reusable biocatalysts for the production of industrially important ester compounds, contributing to the development of more sustainable biotechnological processes.

## Introduction

Enzymatic biocatalysis has proven to be highly effective alternative to traditional chemical catalysis, mainly due to its high catalytic efficiency under mild reaction conditions, particularly at low temperatures, as well as its remarkable selectivity and specificity. In addition, enzymes are renewable catalysts and are generally regarded as environmentally acceptable when compared to conventional chemical catalysts [1, 2]. The global food enzymes market generated approximately US$13,938.9 million in 2024 and is expected to reach US$24,672.1 million by 2033, with an estimated compound annual growth rate of 6.6% between 2025 and 2033 [3].

Among commercial enzymes, hydrolases (EC 3.1.1.3) are the most widely used biocatalysts, with lipases being particularly valued for their versatility and selectivity [4]. Lipases catalyze the hydrolysis of triacylglycerols and can also promote synthesis reactions in non-aqueous media, which has enabled their application in the synthesis of flavor and fragrance esters, an area of increasing industrial relevance [5, 6]. Among the various enzymatic sources, porcine pancreatic lipase (PPL), derived from animal sources, and *Candida rugosa* lipase (CRL), of fungal origin, are widely recognized as important biotechnological models due to their robustness and high efficiency in catalyzing esterification reactions [7]. These esters are typically produced by the reaction of short- or medium-chain carboxylic acids with alcohols, resulting in compounds with fruity and floral aromas. They are widely employed in the food, cleaning, cosmetic, and hygiene industries, where they play a decisive role in sensorial properties and consumer acceptance [8, 9].

The production of esters, whether from natural sources or by conventional chemical synthesis, involves several challenges related to raw material availability, processing conditions, toxicity, and environmental impact [10]. These factors increase production costs and limit the ability to meet the growing demand for global food additives (flavors, aromas, and fragrances), estimated at US$ 12.8 billion in 2023. In this context, enzymatic esterification has emerged as a greener and more sustainable alternative, benefiting from mild operating conditions and alignment with green chemistry principles [11, 12]. Additionally, enzymatically produced esters can be classified as “green esters” since enzymatic processes typically use milder reaction conditions, generate fewer byproducts, and reduce reliance on hazardous catalysts. This can increase both the commercial value and industrial appeal of these compounds [13]. Numerous review studies have reported advances in enzymatic esterification using short-chain alcohols and organic acids, highlighting the maturity and industrial relevance of this biotechnological approach [14, 15]. In addition to the use of short-chain alcohols and organic acids, several studies highlight that ester synthesis can be carried out in systems with organic solvents, in order to facilitate substrate solubilization and reduce the viscosity of the medium, as well as in solvent-free media, which are more sustainable and offer higher productivity, despite posing challenges related to substrate inhibition and mass transfer [16, 17]. However, enzymatic esterification is a thermodynamically controlled reaction, in which controlling water activity is very important to prevent the equilibrium from shifting toward hydrolysis [18]. Furthermore, previous studies have reported that localized water formation around the enzyme can create a hydration layer, leading to inactivation or inhibition [19].

Despite their advantages, the industrial application of free lipases is often limited by their low stability in the desired medium, which remains a major barrier to their use. According to Homaei [20], even the slightest conformational change induced by the enzyme’s surrounding medium can lead to a considerable decline in activity. Enzymes are inactivated under a variety of conditions, such as extreme temperatures or pH values, physical forces, and aqueous-organic or gas-liquid interfaces. Furthermore, the retention of activity is a strict criterion for the integrity of a protein molecule with low long-term operational stability, due to the gradual loss of activity during storage or use and the difficulties associated with recovery and reuse. These limitations significantly restrict their economic feasibility. Enzyme immobilization is therefore a key strategy for overcoming these disadvantages, as in this process the enzyme is bound to an inert (organic or inorganic) and insoluble material that can increase enzyme stability, enable reuse, facilitate recovery, and improve process control [21–23]. Immobilization not only stabilizes the enzyme against denaturation but may also help control the microenvironmental reaction, mitigating the negative effects of water accumulation on thermodynamic equilibrium. Activated carbon is particularly well-suited for this purpose because its predominantly hydrophobic surface and its high surface area can limit the local accumulation of water around the enzyme, helping to maintain conditions favorable for ester synthesis [4, 18, 24].

Beyond the intrinsic properties of activated carbon, surface functionalization with metallic particles can further improve the performance of immobilized enzymes by providing biocompatibility, low toxicity, enhanced stability, and simplified recovery, particularly when magnetic materials are employed. This approach commonly employs chelating agents, such as iminodiacetic acid (IDA), which provide coordination sites for metal ions, increasing their retention on the activated carbon surface and improving the stability of the immobilization system [25–27]. By forming stable metal-ligand complexes, IDA helps minimize iron leaching while maintaining accessible metal sites for specific interactions with the enzyme’s protein structure. As a result, the functionalized support may exhibit higher immobilization efficiency and improved enzyme retention during successive catalytic cycles. Iron oxide (Fe₃O₄) magnetic particles are among the most widely used materials, typically synthesized by co-precipitation of Fe²⁺ and Fe³⁺ ions, and are known for their ability to interact strongly with enzymes [27–29]. Among the materials employed for enzyme immobilization, Fe₃O₄ particles have received considerable attention due to their high surface area, magnetic recoverability, and potential to enhance enzyme-support interactions, facilitating catalyst recovery and operational reuse. Nevertheless, variations in reaction conditions may lead to iron leaching, which can compromise enzyme stability and catalytic performance [25, 30].

In addition to these methods, the surface of charcoal can be modified using a natural agent, genipin, which can be extracted from Gardenia (*Gardenia jasminoides*) or green genipap (*Genipa americana* L). Genipin is a bifunctional and low-toxicity natural cross-linking agent that has attracted considerable attention for enzyme immobilization applications. Its ability to react spontaneously with primary amine groups on both enzymes and support materials enables the formation of stable covalent linkages, which can enhance enzyme stability and increase the structural rigidity of the immobilized biocatalyst [31, 32]. In activated carbon-based systems, the introduction of amino groups onto the support surface provides reactive sites for genipin attachment, thereby enabling the formation of cross-linked networks that enhance enzyme retention and operational stability within the immobilization matrix [33]. Since genipin is natural, it provides improvements in enzymatic properties, such as increased activity, stability, and resistance to chemicals [34, 35]. Although lipases have been extensively studied, the immobilization of lipases on activated carbon functionalized with genipin and metallic particles, particularly in a combined approach, has not yet been reported. Thus, the integration of these modification strategies, including the combined use of genipin, IDA, and iron ions, was proposed to investigate potential synergistic effects related to enzyme-support interactions, structural stabilization, catalyst recovery, and operational performance across successive esterification cycles. In this context, the present study aimed to investigate the effect of different surface modifications - genipin, IDA associated with metallic particles, and the combination of genipin with metallic particles - on activated carbon for the immobilization of porcine pancreatic lipase and *Candida rugosa* lipase, as well as to evaluate their application in the synthesis of ethyl lactate as a model esterification reaction.

## Materials and Methods

### Materials

Activated carbon was synthesized in previous studies [24] using sisal shredding waste provided by Apaeb Sisal, a company located in the municipality of Valente, Bahia. The synthesis was performed using chemical activation with phosphoric acid at an impregnation ratio of 2.5:1 (activating agent mass/precursor dry mass) and a carbonization temperature of 700 °C for 86 min. Table 1 shows the main activated carbon properties determined by Nascimento et al. [24].

**Table 1:** Yield, Ash, Zero charge pH (pHpcz), Specific surface area (Sg); Pore diameter (Dp); Mesopore volume (V_meso_), Micropore volume (V_micro_), and Total pore volume (V_total_) of activated carbon

Yield (%)	Ash (%)	pH_ZCP_	Sg (m^2^ g^− 1)^	Dp* (nm)	V_meso_ (cm^3^ g^− 1^)	V_micro_ (cm^3^ g^− 1^)	V_total_ (cm^3^ g^− 1^)
45.61	7.89	5.83	600	5.68	0.474	0.089	0.602

* Maximum pore size distribution; Nascimento et al. [24]

The concentrated genipin extract (containing 6.46 mg.mL^− 1^ of genipin) was obtained as described by Santos et al. [34], extracted from the mesocarp of unripe genipap fruit with 70% ethanol (C_2_H_5_OH), and subsequently concentrated by rotary evaporation. The concentration was determined by HPLC using a standard genipin calibration curve, and the extract was used directly without further purification. Lipase (CAS: 9001-62-1) from Porcine Pancreas (PPL) type II (≥ 125 units/mg protein) and Lipase from *Candida rugosa* (CRL) type VII (≥ 700 units/mg solid) were obtained from Sigma-Aldrich Corporation. All other chemicals used were of analytical grade and purchased from Sigma-Aldrich, Exodus Scientific, and Synth.

### Functionalization of Activated Carbon

The surface modification was performed using different modifying agents, including genipin and iron salts (Fe^2+^ and Fe^3+^). The functionalization with magnetic particles was also investigated using iminodiacetic acid (IDA) as a chelating agent and genipin as a cross-linking agent.

#### Functionalization with Genipin

Surface modification with genipin was performed as described by Santos et al. [34]. First, 10 g of AC was mixed in an amine solution (2.5% v/v) containing 0.85 mL of ethylenediamine 99% (C_2_H_4_(NH_2_)_2_) and 32.5 mL of acetone (CH_3_(CO)CH_3_). This mixture was manually stirred for 10 min and left to rest for 24 h to obtain amino-functionalized activated carbon.

The amino-functionalized AC was mixed with a genipin solution containing 33.5 mL of genipin extract and 33.5 mL of acetone under magnetic stirring at 85 rpm for 30 min. After the agitation, the sample was dried at 60 °C for 24 h. After that, the functionalized activated carbon was washed with distilled water to remove any unbound chemical compounds and dried at 60 °C for 6 h. Finally, the genipin-functionalized activated carbon (GAC) was sieved (40 mesh) and packaged in hermetic packaging.

#### Functionalization with Magnetic Particles

The surface modification using iron salts was carried out by co-precipitating Fe^2+^ and Fe^3+^ ions to incorporate magnetic particles on the activated carbon surface and create cross-links with enzymes. For this, two methods were employed: functionalization with iminodiacetic acid (IDA) as a chelating agent and genipin as a cross-linking agent, followed by incorporation of metallic particles.

i. Iminodiacetic acid as a chelating agent

To incorporate iminodiacetic acid (IDA) as a chelating agent, 3 g of AC were mixed with 20 mL of 1 M sodium carbonate (Na_2_CO_3_) under magnetic stirring for 1 h. Then, 20 mL of 0.5 M iminodiacetic acid 98% (HN(CH_2_COOH)_2_) was added, and the pH was adjusted to 11 with 1 M sodium hydroxide (NaOH). The mixture was stirred at 20 rpm and 40 °C for 14 h in a BOD incubator. After this period, the activated carbon was washed with an acetic acid solution (1% v/v) until the water became neutral.

The metallic particles were synthesized following Mohan et al. [36] and Santos et al. [34] through the co-precipitation of ferrous sulfate (Fe^2+^) and ferric chloride (Fe^3+^) in an alkaline solution. For this, 5 g of CA were mixed with 250 mL of ultrapure water while stirred magnetically (100 rpm) for 10 min. The iron salts solution was prepared by mixing 200 mL of a solution containing 0.067 mol of trivalent iron (FeCl_3_.6H_2_O), 200 mL of a solution with 0.033 mol of bivalent iron (FeSO_4_.7H_2_O), and 2.5 mL of 37% HCl. The ferrous sulfate and ferric chloride solutions were added to the AC and stirred at 70 °C for 15 min. After this step, 500 mL of 4 M sodium hydroxide (NaOH) was added under magnetic stirring to precipitate the particles for 2 h. The precipitate was filtered and washed with ultrapure water under vacuum until the pH reached neutrality (pH ~ 7.0). After washing, the magnetic activated carbon with iminodiacetic acid as a chelating agent (MAC) was dried at 60 °C for 24 h.

ii. Genipin as a cross-linking agent

Activated carbon cross-linked with genipin and functionalized with magnetic particles (GMAC) was prepared according to the methods described in 2.2.1 and 2.2.2 (i), respectively.

### Supports Characterization

All the supports produced were characterized by Fourier Transform Infrared Spectroscopy (FTIR), point of zero charge (pH_pcz_), Porosity, BET surface area, X-Ray Diffraction (XRD), Scanning Electron Microscopy (SEM), and Energy-Dispersive Spectroscopy (EDS).

The functional groups of the samples were analyzed by FTIR using the attenuated total reflectance (ATR) technique in the infrared region (4000–600 cm⁻¹) with an Agilent Cary 630 FTIR spectrophotometer (Agilent Technologies Inc., Santa Clara, CA, USA). The point of zero charge (pH_pcz_) was obtained as described by Kuśmierek et al. [37]. The specific surface area was measured by the Brunauer–Emmett–Teller (BET) Equation [38]. The pore size distribution was obtained through the desorption isotherm using the Barrett–Joyner–Halenda (BJH) method, and the micropore volume was achieved from the t-plot analysis according to the adsorption isotherms [39] on a Micrometrics ASAP 2420 equipment (Georgia, USA) using approximately 0.20 g of sample at −196.15 °C (liquid nitrogen temperature). SEM was used to determine surface morphology for the samples using the JSM − 6610 (Jeol, Tokyo, Japan), equipped with EDS Thermo Scientific (NSS Spectral Imaging, USA) (Denton Vacuum LLC, Moorestown, New Jersey, USA), equipped with the carbon accessory (Quanta 250, Fei Company, Waltham, MA, USA). X-ray diffractograms were obtained on a Rigaku Miniflex diffractometer (Tokyo, Japan) equipped with a stationary copper tube as the X-ray source (Kα1.2; λ = 1.5418 Å; approx. 8.0 keV). The generator tension was 30 kV/15 mA. Support crystallinity was evaluated at room temperature in 2theta (2θ) varying from 10º to 90º with a speed rate of 4.2º/min.

### Enzyme Immobilization

Lipase from Porcine Pancreas (PPL) and *Candida Rugosa* (CRL) were immobilized on all produced supports. Initially, 0.1 g of support and 5 mL of lipase solution: 6000 mg.L^− 1^ for PPL or 2000 mg.L^− 1^ for CRL in sodium acetate buffer (0.1 M, pH 5.0) were stirred on an orbital shaker at 150 rpm and 30 °C for 2 h [40]. The samples were centrifuged (3500 rpm, 5 min), and the supernatant was collected to measure protein concentration using the Bradford method with BCA as the reference protein [41]. The immobilization capacity was calculated using Eq. 1, and the immobilization efficiency (E%) was determined as the ratio of the immobilized lipase mass to the initial lipase mass.

1 $$\:{C}_{imo}=\frac{v\:({C}_{i}-C)}{{m}_{a}}$$

Where: C_Imo_ is the immobilization capacity (mg.g^− 1^); v is the volume of lipase solution (mL); C_i_ is the initial lipase concentration (mg.L^− 1^); C is the lipase concentration at equilibrium (mg.L^− 1^); m_a_ is the adsorbent mass (g).

Preliminary tests were conducted to determine which concentrations would be required to achieve approximately equal initial enzymatic activities without compromising the comparison between results, since the lipases had different purities.

### Hydrolytic Activity

Lipolytic activity was determined using the olive oil emulsion hydrolysis method, following the methodology described by Nascimento et al. [24]. One unit of activity was defined as the amount of enzyme that releases 1 µmol of fatty acid per minute of reaction under the test conditions. Enzyme Activity Yield (%) was calculated by the ratio between immobilized and free enzyme activities.

### Ethyl Lactate Synthesis

The synthesis of ethyl lactate, used as a model esterification reaction, was carried out following the method described by Oliveira et al. [29], with slight modifications. The substrate was prepared by mixing lactic acid (100 mM) and ethanol (300 mM) in hexane solvent. For each 0.5 g of the derivative, 5 mL of the reaction mixture was added, and the mixture was incubated in a shaking bath at 40 °C and 200 rpm for 4 h. The lactic acid content was determined after the reaction by titration (30 mM NaOH) until pH 11 was reached. Control tests were performed in the absence of the catalyst and using only enzyme-free support, under the same operating conditions as in the catalytic experiments. The results were presented as esterification yield (Y%), calculated using Eq. 2.

2 $$\:Y\:\left(\%\right)=\:\frac{\left({C}_{0}-\:{C}_{f}\right)}{{C}_{0}}\:\times\:100$$

where *C*_0_ is the initial concentration, and *C*_f_ is the final concentration of lactic acid (mM).

### Operational Stability

The derivatives’ operational stability was evaluated through their reuse in multiple cycles of esterification reactions between lactic acid and ethyl alcohol, as described in Sect. 2.6. After each cycle, the derivatives were centrifuged and all supernatant was removed. The derivatives were then washed in two steps: first with sodium acetate buffer (0.1 M, pH 5.0) and then with hexane, followed by centrifugation to remove the solutions before starting a new reaction (as described in Sect. 2.6). Stability was determined using the yield for each cycle as a parameter.

### Statistical Analysis

The data was evaluated by analysis of variance (ANOVA) with the F test followed by the Tukey test for comparison between means (*p* < 0.05) using SAS Studio and Sigmaplot software. All assays were performed in triplicate.

## Results and Discussion

### Characterization of Activated Carbons

#### Fourier Transform Infrared Spectroscopy (FTIR)

**Fig. 1:**
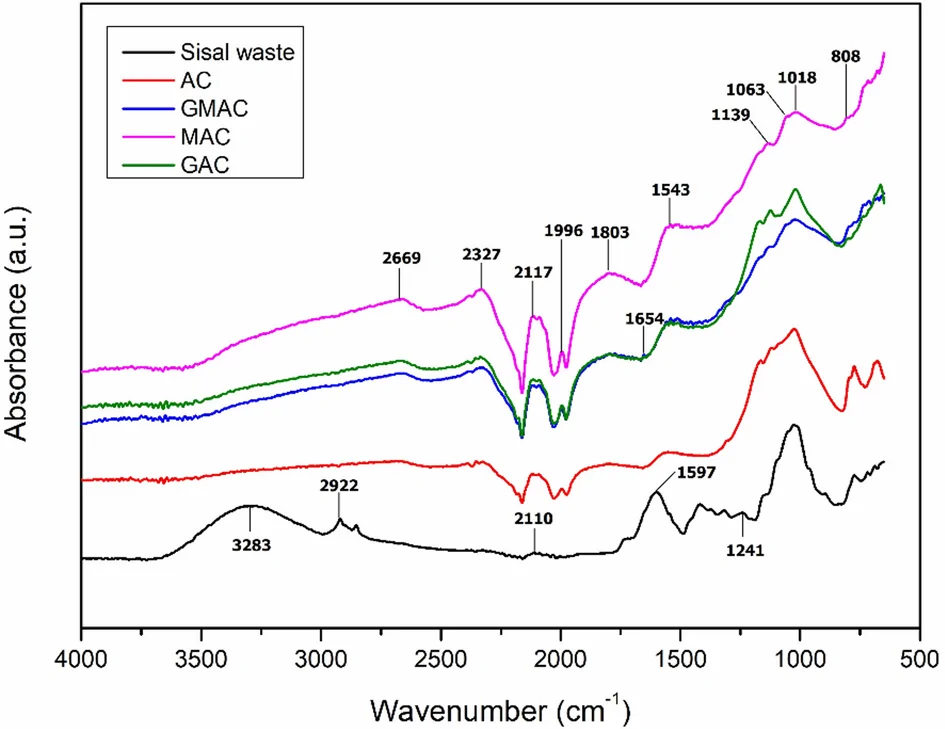
FTIR of sisal waste, activated carbon (AC), and activated carbon functionalized with genipin (GAC), IDA + metal (MAC), and genipin + metal (GMAC)

Fourier transform infrared spectroscopy (FTIR) was used to determine the functional groups present in sisal waste, activated carbon (AC), and AC modified with genipin (GAC), IDA + metal (MAC), and genipin + metal (GMAC), as shown in Fig. 1.

Sisal waste exhibited functional groups associated with lignocellulosic content. These include a band at 3283 cm^− 1^ referred as the hydroxyl group (OH) in the cellulose structure; the band at 2922 cm^− 1^, which represents the vibration of methyl group (C–H) from cellulose, hemicellulose, and lignin; 1597 cm^− 1^ is attributed to the C = O bond in carboxymethyl cellulose; 1241 cm^− 1^ is associated to the guaiacyl groups; and the wavelength at 1027 cm^− 1^ is related to the stretching of the glycosidic bonds (C–O and C–O–C) of lignin, cellulose and hemicellulose [42, 43].

During the synthesis of activated carbon, chemical activation and pyrolysis led to the thermal decomposition of some functional groups (3283 cm⁻¹, 2922 cm⁻¹, and 1241 cm⁻¹), while new groups were formed as expected and reported in the literature [24]. It was verified that the band at 2330 cm^− 1^ corresponds to the methyl group; 2110 cm^− 1^ is attributed to the stretching of C–O bond from ketone groups; the wavelength at 1996 cm^− 1^ is associated to the carboxylic acid anhydrides groups; 1794 cm^− 1^ is attributed to C = O vibrations of ketone, aldehyde, lactone or carboxylic groups; the band at 1162 cm^− 1^ is related to the C–O vibrations in tertiary alcohols; and 1070 cm^− 1^ represents the phosphate ester bonds from the activation with phosphoric acid (P^+^–O^−^ and P–O–P) [44, 45].

An increase in peak intensity was observed for all functionalized supports compared to CA, especially metalized activated carbon (MAC), thus indicating that the modification of the support surface was effective. The presence of amine groups suggests that ethylenediamine was successfully incorporated onto the activated carbon surface via interactions with oxygen-containing groups, such as carboxylic groups generated during activation, thereby creating anchoring sites for subsequent genipin cross-linking and surface modification [40]. Additionally, new vibrations were observed in all functionalized matrices, including bands at 2669 cm^− 1^ and 1543 cm^− 1^, attributed to methyl and amine groups, respectively. Regarding adsorbents functionalized with magnetic particles, the incorporation of metal and IDA (MAC) showed a higher peak intensity than that of metal and genipin (GMAC), due to stronger interaction between the functional groups and Fe_3_O_4_ via a chelating agent. This may be due to the strong binding ability of iminodiacetic acid functional groups for most metal ions [25, 46].

#### Point of Zero Charge (pH_PCZ_)

The surface charge of the carbon materials was determined through the point of zero charge (pH_PCZ_). Activated carbon (AC) obtained from sisal residues showed a pH_PCZ_ = 5.83. This low value is associated with the presence of acidic groups generated by chemical activation with phosphoric acid, as well as the dissociation of acidic surface oxygen complexes [40].

After modification, an increase in pH_PCZ_ was observed in the functional supports compared to CA. For the support functionalized with genipin (GAC), the pH_PCZ_ was 6.54, an increase attributed to surface amination performed to incorporate genipin. In the supports functionalized with metal, the values were even higher: MAC (pH_PCZ_ = 8.13) and GMAC (pH_PCZ_ = 7.94). This increase may be related to the precipitation of metal ions, a phenomenon typically observed in alkaline media [47]. The difference between GMAC and MAC may be due to the higher iron oxide content on the surface in MAC, resulting from chelation by iminodiacetic acid [25]. On the other hand, the acid groups of genipin may have contributed to the slightly lower pH_PCZ_ value observed for GMAC compared to MAC [48].

The high pH_PCZ_ values of the functionalized supports are relevant for enzyme immobilization. During the process, carried out at pH 5.0, these supports will present a positive surface charge, while Porcine Pancreatic Lipase (PPL), with PI ≈ 4.9 [49], and *Candida rugosa* lipase (CRL), with PI ≈ 4.2 [50], will have neutral or negative charges. This charge difference may favor electrostatic interactions, contributing to more efficient immobilization.

#### Texture Properties

**Fig. 2:**
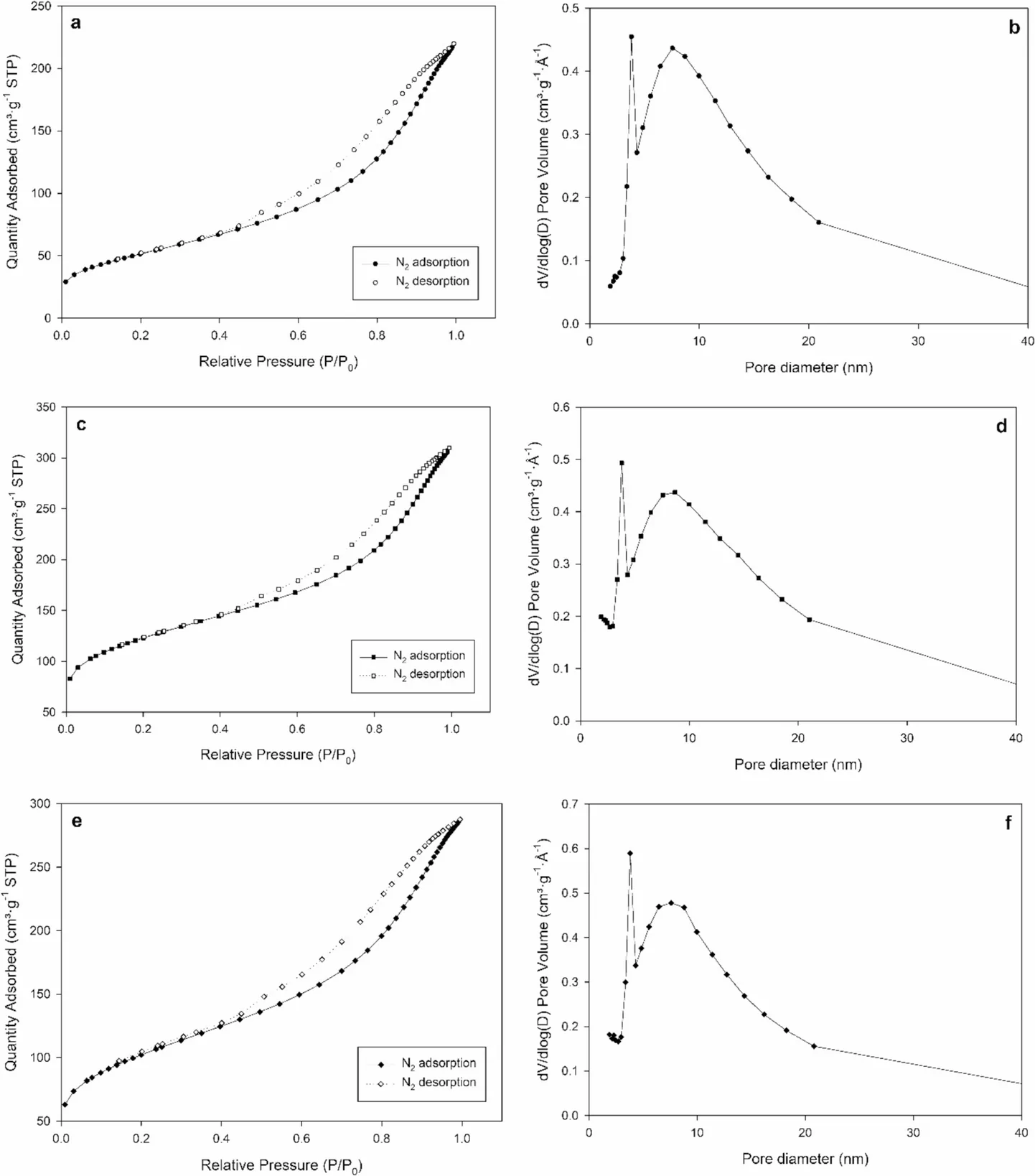
Nitrogen adsorption-desorption isotherms and pore distribution of activated carbon functionalized with genipin (GAC) (**a** and **b**), IDA + metal (MAC) (**c** and **d**), and genipin + metal (GMAC) (**e** and **f**)

Figure 2 shows the nitrogen (N_2_) adsorption-desorption isotherms and pore size distribution of activated carbon functionalized with genipin (GAC), IDA + metal (MAC), and genipin + metal (GMAC)

All the functionalized supports exhibited Type IV isotherms according to the IUPAC classification [51]. Hysteresis refers to the capillary condensation in mesopore structures, and it may exhibit a wide variety of shapes, such as Type H1 of the hysteresis loop. This loop type is often associated with porous materials that exhibit agglomerates or compacts of regular arrays and narrow pore-size distributions. The first nitrogen slope was observed in the low-pressure region (P/P_0_ < 0.5), attributed to the monolayer coverage. On the other hand, at P/P_0_ > 0.5, the multilayer adsorption began. It was also verified that MAC adsorbed the highest N_2_ volume (309.89 cm^3^ g^− 1^ STP) among the other matrices, except for activated carbon (395.63 cm^3^ g^− 1^ STP), suggesting a greater pore volume and surface area available for adsorption. These observations can be confirmed by analyzing the pore-size distribution (Fig. 2b, d, and f) and the texture properties of the supports (Table 2).

**Table 2:** Texture properties of activated carbon functionalized with genipin (GAC), IDA + metal (MAC), and genipin + metal (GMAC)

Sample	Sg (m^2^ g^− 1^)	Dp (nm)^a^	V_meso_ (cm^3^ g^− 1^)	V_micro_ (cm^3^ g^− 1^)	V_total_ (cm^3^ g^− 1^)
GAC	187	6.82	0.3317	0.0026	0.3393
MAC	433	6.04	0.3850	0.0602	0.4694
GMAC	366	5.79	0.3847	0.0257	0.4369

Sg – BET surface area; Dp – average pore diameter; a – maximum pore size distribution; Vmeso – mesopore volume; Vmicro – micropore volume; Vtotal – total pore volume

The functionalized activated carbons exhibited a pore distribution with peaks at 3.8 and 10 nm in diameter, indicating they are micromesoporous. In addition to the standard AC (0.769 cm³.g^− 1^), GMAC showed the highest pore volume (0.589 cm³.g^− 1^). The adsorption phenomenon is influenced by the close relationship between mesopores and micropores in materials, as mesopores facilitate the diffusion of compounds into micropores, where adsorption occurs [49, 52]. Additionally, Table 2 shows the texture properties of supports functionalized with genipin (GAC), IDA + metal (MAC), and genipin + metal (GMAC).

Regarding the activated carbon properties (Table 1), all functionalized supports showed decreases in surface area and pore volume, confirming the surface changes. This behavior may be due to the insertion of the chelating/crosslinking agent and magnetic particles into the pores during the functionalization process, leading to pore blockage [53]. These findings show that the BET area depends on pore characteristics, especially the micropores. As pore volume decreases, surface area decreases as well. However, the modifications led to an increase in the pore diameter, attributed to smaller pores being obstructed by the functionalizing agents, resulting in a higher average pore diameter [34].

Genipin-functionalized AC demonstrated the lowest BET area and pore volume among the functionalized adsorbents. This demonstrates a 68.8% reduction in the surface area compared to unfunctionalized activated carbon. Genipin is a crosslinking agent that forms stable crosslinked structures, which can lead to greater pore blocking [54]. This can be verified by observing the genipin-iron oxide interaction in GMAC, resulting in lower pore obstruction and higher surface area (39% reduction compared to the original matrix).

Although the BET area was reduced by 27.8%, the highest surface area and pore volume between the functionalized activated carbons was observed for MAC. The higher values for both metal-functionalized adsorbents suggest that most iron particles were incorporated into the surface, and the chelating agent (MAC) was more effective in retaining iron oxide on the surface than the crosslinking agent (GMAC). The greater the texture parameters, the more efficient lipase immobilization tends to be, since they provide a larger area for bonding between the enzyme and the support [40].

**Fig. 3:**
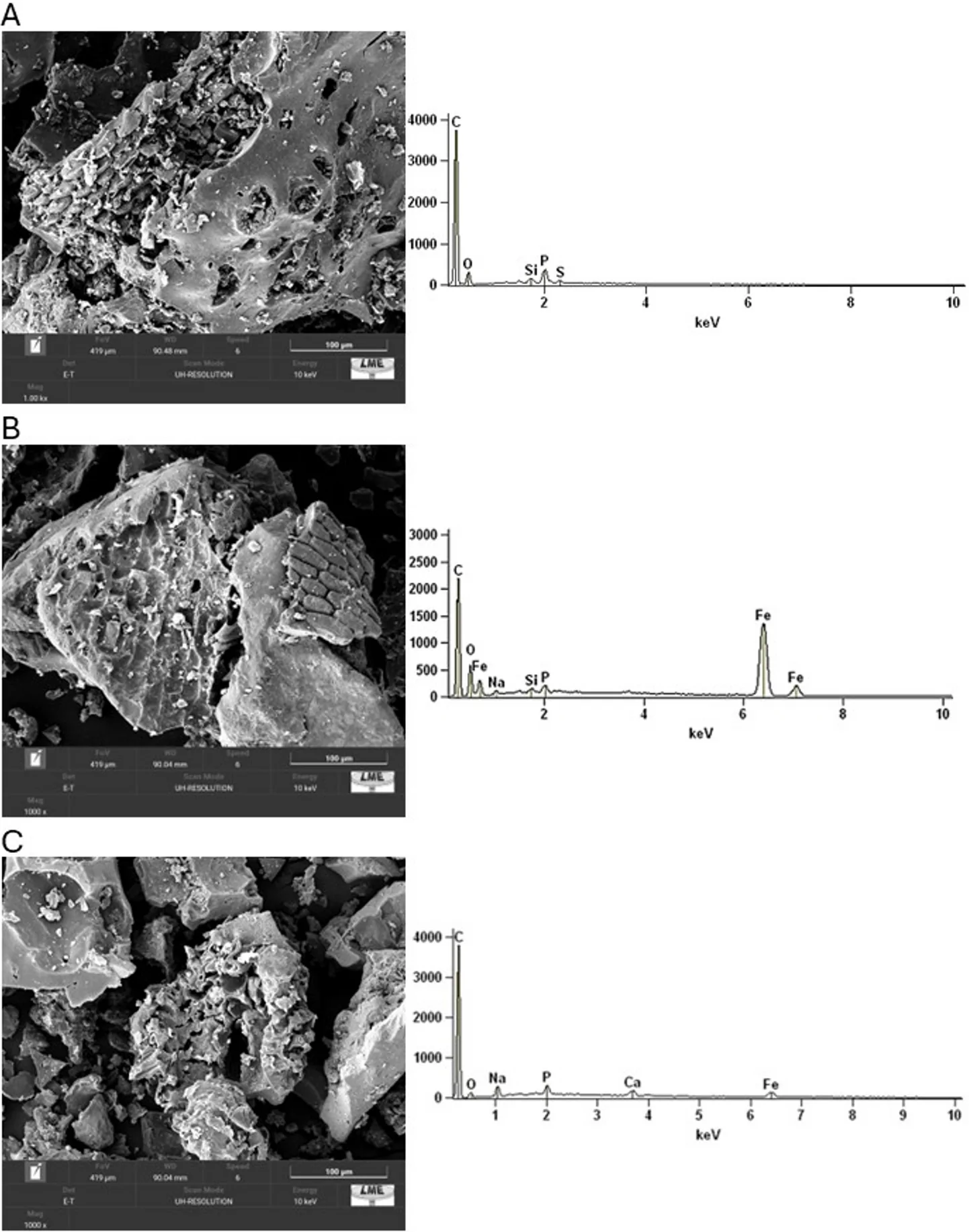
Scanning Electron Microscopy (SEM) and energy dispersive spectroscopy (EDS) of activated carbon functionalized with genipin (GAC) (**a**), IDA + metal (MAC) (**b**), and genipin + metal (GMAC) (**c**)

The texture changes on functionalized activated carbons were also observed through Scanning Electron Microscope (SEM) (Fig. 3). The surface morphology of functionalized supports exhibits a rough texture and a porous structure with varying pore sizes and shapes. This statement aligns with the analyses of area and pore distribution. Figure 3 also confirms that the functionalization was successful, as the structure became smoother, with smaller pores resulting from obstruction by the modifying agents.

The chemical composition of the support surfaces was determined by energy-dispersive X-ray spectroscopy (EDS) analysis, performed in conjunction with SEM. The results (Fig. 3) show that for both modified supports, carbon is the element with the highest concentration. Some impurities, such as silicon, sodium, and sulfur, were also detected, as well as phosphorus resulting from chemical activation with phosphoric acid. When comparing the modifications using iron ions, it is noted that in the modification using a chelating agent and iron ions (Fig. 3-B), it was possible to detect peaks associated with iron on the carbon surface, while in the modification using genipin and iron ions (Fig. 3-C), a small iron peak was observed, indicating that genipin does not have a good ability to interact with iron ions and keep them on the surface of the support. Therefore, we can highlight that the use of the chelating agent (IDA) was able to retain iron ions on the surface of activated carbon with greater efficiency.

**Fig. 4:**
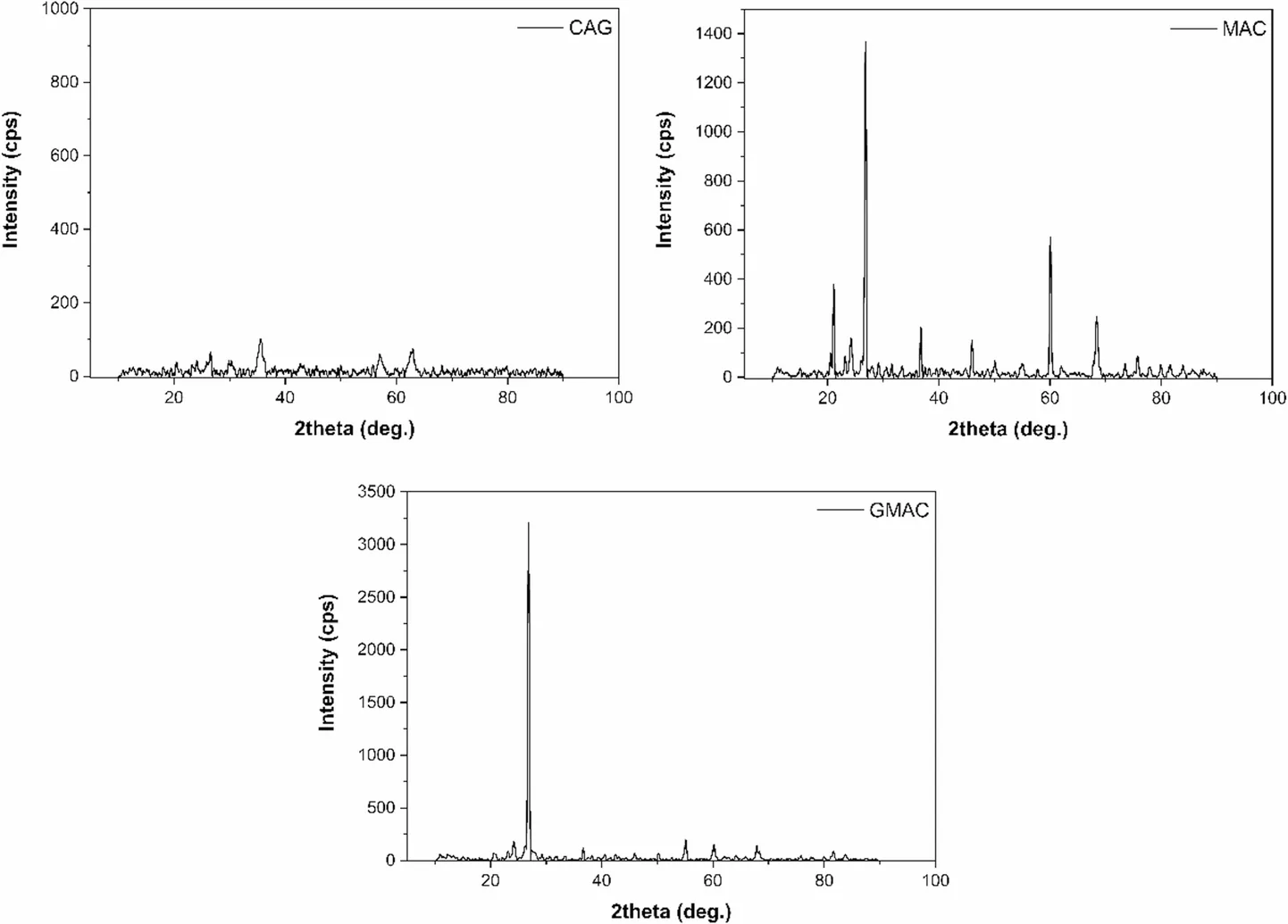
X-ray diffraction (XRD) patterns of activated carbon functionalized with genipin (GAC) (**a**), IDA + metal (MAC) (**b**), and genipin + metal (GMAC) (**c**)

Complementing the morphological (BET) and elemental (SEM/EDS) analyses that revealed significant changes in the surface structure of the modified materials, X-ray diffraction (XRD) was used to evaluate the crystalline structure of the materials, especially for the supports that underwent modifications using iron ions. The diffractograms for the modified supports can be seen in Fig. 4.

When comparing the diffractograms (Fig. 4), it can be observed that activated carbon functionalized exclusively with genipin (GAC) (Fig. 4a) does not present defined crystallinity peaks, exhibiting an essentially amorphous profile. This behavior was expected, since genipin does not induce the formation of crystalline regions in the support, acting only as a surface functionalization agent [34].

**Fig. 5:**
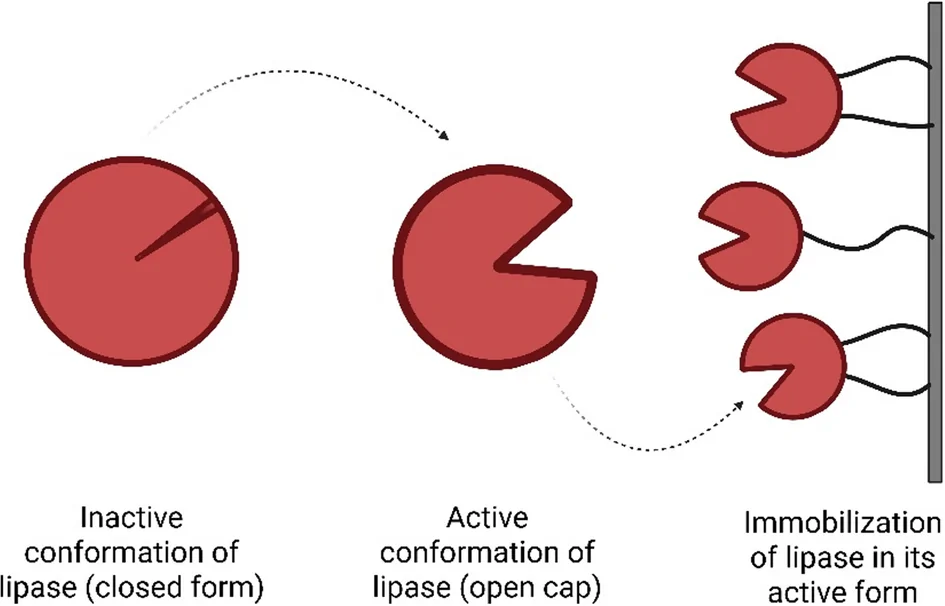
Scheme of interfacial activation of lipase and its immobilization on a hydrophobic support

For the sample containing IDA and metal (MAC) (Fig. 5b), crystalline peaks appear at specific 2θ positions, attributed to the presence of the incorporated metal and the formation of metal complexes on the surface of the carbon, indicating the development of partially ordered regions, suggesting an interaction between the metal and the functional groups of the support. Among the peaks formed at 2θ, we can highlight: 26.8°, corresponding to hematite; 36.7° and 45.9°, attributed to magnetite diffraction; and 60.1° and 68.4°, both referring to the crystalline diffraction of iron oxide [55, 56]. In contrast, the material functionalized with genipin and metal (GMAC) (Fig. 4c) showed the formation of only one prominent crystalline peak (2θ: 26.8), which may be associated with the presence of the inserted metal. When analyzing the results obtained in EDS and XRD together, they corroborate the results, indicating that both genipin and IDA were able to retain the metal particles on the surface of the support; however, the chelating agent IDA provided greater retention of the magnetic particles, favoring a more homogeneous distribution and with more metal groups on the surface of the modified activated carbon.

### Lipase Immobilization

#### Effect of Lipase Source on Immobilization

The effect of lipase source for immobilization on different activated carbons was investigated based on their Immobilization Efficiency (%), Enzyme Activity (U), and Enzyme Activity Yield (%) as shown in Table 3.

**Table 3:** Effect of activated carbon functionalization on lipase immobilization

Sample	Porcine Pancreas Lipase (PPL)			Candida rugosa Lipase (CRL)		
	Immobilization Efficiency (%)	Enzyme Activity (U)	Enzyme Yield (%)	Immobilization Efficiency (%)	Enzyme Activity (U)	Enzyme Yield (%)
GAC	89.32 ± 0.012 ^B, b^	31.3 ± 2.851 ^A, b^	53.64 ± 4.887 ^A, b^	98.17 ± 0.002 ^A, a^	32.2 ± 0.917 ^A, b^	54.67 ± 1.556 ^A, c^
MAC	99.43 ± 0.002 ^A, a^	46.9 ± 1.652 ^A, a^	80.38 ± 2.832 ^A, a^	98.76 ± 0.002 ^A, a^	47.3 ± 0.346 ^A, a^	80.30 ± 0.588 ^A, b^
GMAC	87.41 ± 0.018 ^B, b^	48.1 ± 3.822 ^A, a^	82.43 ± 6.551 ^B, a^	97.90 ± 0.002 ^A, a^	51.6 ± 0.520 ^A.a^	87.61 ± 0.882 ^A, a^

*Means followed by the same lowercase letter in the support type and uppercase letter in the lipase source do not differ significantly from each other according to Tukey’s test (*P* < 0.05)

Lipase from porcine pancreas had the highest Immobilization Efficiency for MAC, while the highest Enzyme Activity and Enzyme Yield were found for GMAC. However, Tukey’s test showed no significant difference between GMAC and MAC. For *Candida rugosa* lipase, no significant difference in immobilization efficiency was observed, with the highest yield achieved with GMAC.

The differences in immobilization and activity parameters across supports may be due to the strength and orientation of the lipase attachment. Although immobilization is suitable for all materials, the biocatalyst can bind with the active site blocked or substrate diffusion restricted. Lipase has a specific catalytic property called interfacial activation, which occurs at the oil-water interface, allowing the enzyme to adopt two configurations: the closed, inactive conformation and the open, active conformation. This mechanism has a mobile hydrophobic α-helix polypeptide chain that acts as a “lid” and blocks the active site from the reaction medium. However, the lid opens in the presence of a hydrophobic interface and aids the substrate-active site interaction. Thus, the biocatalyst activity is influenced by the immobilization on hydrophobic surfaces such as activated carbon [57–59]. This mechanism of action can be seen in Fig. 5. Furthermore, the functionalizing agents tend to incorporate the enzyme through strong bonds, changing the lipase conformational structure and decreasing its flexibility [60, 61]. The formation of multipoint covalent bonds, such as Schiff bases between amino groups on the enzyme surface and carbonyl/aldehyde groups on the activated support, increases structural rigidity, protecting the enzyme against denaturation, although it may slightly restrict the conformational flexibility required for certain catalytic transitions [62, 63].

Comparing the two enzyme sources, *Candida rugosa* lipase showed significantly higher efficiency (> 97% across all supports). Since the molecular weight of PPL is around 50 kDa [64] and CRL is around 60 kDa [65], the immobilization process of CRL was probably more influenced by interactions on the activated carbon surface than inside the pores. For the other parameters, CRL also had the highest values, but they did not differ significantly from each other. This can be attributed to the higher purity of CRL, since PPL is supplied as a crude porcine pancreas extract containing other hydrolases as contaminants, such as esterases, amylases, and proteases [66]. For instance, experiments were conducted at different protein concentrations (PPL = 6000 mg L^− 1^ and CRL = 2000 mg L^− 1^) to achieve comparable lipolytic activity.

Moreover, each source has its own specificity and selectivity to the substrate. Regarding regiospecificity, PPL is sn-1,3-specific and hydrolyzes ester bonds at the sn-1 and sn-3 positions, releasing free fatty acids and 2-monoacylglycerol. On the other hand, CRL is nonspecific and randomly hydrolyzes all triglyceride positions. This results in a mixture of products, including free fatty acids, monoacylglycerols, diacylglycerols, and glycerol [2]. Lipases also show selectivity for different types of fatty acids, such as *Candida rugosa* lipase, which preferentially recognizes oleic acid substrates [57]. The olive oil used as a substrate in our work has a high oleic acid content (56–84% of total fatty acids), making it suitable for the CRL catalytic mechanism [67].

#### Effect of Surface Modification on Lipase Immobilization

Surface functionalization of activated carbon significantly influenced the immobilization parameters of different lipase sources, as shown in Table 3, with variations observed in Immobilization Efficiency (%), Enzyme Activity (U), and Enzyme Activity Yield (%). The highest immobilization efficiency was achieved by MAC (> 98.76%), followed by GAC (> 89.32%) and GMAC (> 87.41%). On the other hand, GMAC exhibited the highest enzymatic activity and activity yield, followed by MAC and GAC, respectively.

Among the functionalized matrices, the genipin-functionalized support showed the lowest immobilization parameters for both enzymes. Immobilization efficiency was the only variable that showed a significant difference according to Tukey’s test; however, no significant difference was found between the supports. Despite this, GAC showed higher values than activated carbon from sisal waste for PPL (E% = 88.73, U = 23.6, R% = 33.33) and CRL (E% = 99.55, U = 30.9, R% = 52.46) [24].

Even with a low surface area, genipin-carbon showed a high immobilization efficiency, suggesting that immobilization occurred via spacer arms inserted into the surface. The functionalization method involves carbon surface amination followed by genipin incorporation via amino-group attack on the carbonyl of the genipin ester (C-11), forming a stable amide bond. The initial interaction between ethylenediamine and activated carbon is proposed to occur through reactions and strong interactions with oxygen-containing surface functionalities (carboxyl and carbonyl groups), creating anchoring points for subsequent genipin immobilization. This pre-activation step may also generate spacer arms that reduce steric hindrance between the enzyme and the rigid carbon matrix [63]. Due to its bifunctional properties, genipin can also bind to proteins via ion-exchange, covalent, or hydrophobic interactions. Under low pH conditions, the primary amines of the amino acid side chains (such as lysine, hydroxylysine, and arginine) make a nucleophilic attack on the C-3 of genipin. This attack opens the genipin dihydropyranic ring and generates an intermediate aldehyde. Subsequently, the intermediate aldehyde group undergoes an aldol condensation with the secondary amines, resulting in a new heterocyclic compound [68, 69]. The formation of Schiff base linkages between aldehyde groups formed during genipin activation and lysine residues present on the enzyme surface may promote multipoint covalent immobilization. As highlighted by Bolivar et al. [62], these strong enzyme-support interactions are desirable for enhancing biocatalyst stability; however, excessive structural rigidity may restrict the conformational flexibility required for catalysis, which may partially explain the reduction in recovered activity after immobilization.

Regarding the low enzyme activity, the low enzyme yield suggests that decreased biocatalyst mobility following immobilization may have imposed greater restrictions on the substrate. Since this is a novel immobilization method for lipases, it is very likely that the potential of this chemical is not yet fully exploited. Therefore, further studies should be conducted to evaluate optimal modification and immobilization conditions to increase process efficiency. Furthermore, tests can be performed by incorporating pure genipin to enhance surface properties [70].

Metal-functionalized carbon (MAC) was highly efficient for immobilization (E > 98%), with a significant difference, according to the Tukey test, for Porcine Pancreatic Lipase. Since the molecular weight of PPL is lower than that of CRL, the high surface area and pore volume of this support may favor PPL adsorption within the pores. In addition, the high hydrolytic activity observed in both metalized substrates can be attributed to interfacial activation of lipases induced by the hydrophobic and electrical properties of iron oxide particles. Thus, the metal surface may promote the open conformation of the enzyme by interaction with the lipase “lid,” exposing the active site and facilitating substrate access, thereby increasing catalytic efficiency relative to the free enzyme [71].

Furthermore, MAC activity and yield did not differ significantly from GMAC, although the enzymatic values were not the highest. The interaction mechanism between magnetic particles and enzymes is mainly influenced by electrostatic, van der Waals, and hydrophobic forces, followed by covalent bonding between epoxy groups of iron oxide and amino acids (amino, thiol, or hydroxyl groups) of lipase [72, 73]. The presence of metal ions on the surface of activated carbon can also act as Lewis sites, stabilizing intermediate reaction or orienting water molecules toward hydrolysis, which may explain why metallic functionalization is more effective than simple physical adsorption on unmodified activated carbon [74].

GMAC showed the lowest immobilization efficiency, despite having an intermediate surface area. This can be explained by steric hindrance caused by the presence of large molecules on the surface of activated carbon, such as the genipin spacer arm and metal particles [35], which hinder access and binding of the lipase. However, the fraction of the enzyme that successfully bound to the support exhibited the highest activity and yield compared to the free enzyme. This indicates that, although the amount of immobilized enzyme is lower, it remains firmly bound and maintains its conformation, preserving high catalytic activity. This superior stability and performance are directly related to the high point of zero charge (pH_PCZ_) of the metalized supports, which, under the immobilization pH conditions, provides a net positive charge to the surface. This favors strong electrostatic interactions with the negatively charged enzyme surface, promoting an orientation that minimizes active-site blockage and maximizes the biocatalyst’s structural stability [29, 75].

Although influenced by different factors, both iminodiacetic acid and genipin proved effective at retaining magnetic particles on the AC surface when used as chelating and cross-linking agents, respectively. However, GMAC offers a green approach by eliminating a chemical compound from the process and reducing costs by using genipin extract in the modification method. Therefore, genipin has the potential to replace IDA as a binding agent.

For all functionalized activated carbons, high immobilization efficiency was observed despite differences in texture properties. These findings are attributed to differences in charge on the supports and the enzyme under the immobilization conditions. Therefore, immobilization was favored by electrostatic interactions, especially for adsorbents with surface groups that enhance these interactions (MAC and GMAC) [34]. GMAC was influenced by crosslinking (genipin) and electrostatic interactions (iron oxide). Thus, the superiority of metalized supports stems not only from their surface area but also from a synergistic combination of surface chemistry (high pH_PCZ_) and the ability to induce interfacial activation, making them promising supports for biotechnological applications.

### Ethyl Lactate Synthesis

Once lipolytic immobilization proved to be efficient, the derivative was evaluated for the synthesis of aroma esters. The esterification of ethyl lactate using different lipase sources and functionalized activated carbons, as measured by Yield of Esterification (Y%), is presented in Table 4, with all values exceeding 90%. As described by Kasche [18], many enzymatic esterification reactions are thermodynamically controlled, such that the final conversion is governed by the reaction equilibrium rather than by enzyme activity alone, requiring appropriate process design strategies to shift the equilibrium toward ester synthesis. Within this context, the control experiments provide important confirmation of the enzyme’s catalytic role: no ethyl lactate was detected under the evaluated conditions, with zero readings. These results demonstrate that spontaneous esterification between lactic acid and ethanol does not occur and that the support itself exhibits no catalytic activity. Consequently, the ester formation observed in the catalytic assays can be attributed exclusively to enzymatic catalysis, which accelerates the reaction toward equilibrium without altering its thermodynamic limitation.

**Table 4:** Conversion of ethyl lactate ester catalyzed by PPL and CRL (FE) immobilized on activated carbon functionalized with genipin (GAC), IDA + metal (MAC), and genipin + metal (GMAC)

Sample	Conversion (Y%)	
	Porcine Pancreas Lipase (PPL)	Candida rugosa Lipase (CRL)
FE	94.97 ± 0.96 ^B, a^	96.57 ± 0.22 ^A, a^
GAC	91.92 ± 0.69 ^A, b^	91.48 ± 0.07 ^A, b^
MAC	91.44 ± 0.06 ^A, b^	92.88 ± 0.83 ^A, b^
GMAC	94.21 ± 0.76 ^A, a^	92.30 ± 0.04 ^B, b^

*Means followed by the same lowercase letter in the column and uppercase letter in the row do not differ significantly from each other according to Tukey’s test (*P* < 0.05)

For Porcine Pancreatic Lipase (PPL), GMAC showed the highest ethyl lactate conversion yield among the supports, with a conversion efficiency comparable to that of the free enzyme (FE). For *Candida rugosa* lipase (CRL), all supports showed similar conversion yields, but lower than the free enzyme’s conversion capacity. The superior performance of GMAC for PPL compared to the other supports can be attributed to the strong binding of the biocatalyst in a favorable orientation, favoring higher esterification activity.

The free enzyme (FE) exhibited, in some cases, slightly higher conversion due to its greater mobility and the absence of diffusional limitations. Nevertheless, the use of immobilized derivatives offers decisive practical advantages for industrial applications. Unlike the native form, the functionalized supports enable biocatalyst recovery and reuse, reduce contamination of the final product by residual proteins, and enhance thermal stability and tolerance to organic solvents. These characteristics help overcome the high costs associated with the single use of free enzymes and simplify downstream purification steps in the synthesis process [76, 77].

Despite the low hydrolytic activity, GAC achieved high ester conversion, indicating high enzyme specificity for esterification reactions and protection of the active site even after immobilization [77]. As previously mentioned, genipin is a cost-effective and low-toxicity crosslinking agent. Therefore, it can serve as an alternative to conventional functionalization methods, such as glutaraldehyde, and is effective for the synthesis of ethyl lactate [35].

When comparing the two biocatalyst sources, CRL showed the highest FE yield, although there was no significant difference in PPL. On the other hand, PPL reached the highest significant conversion for immobilized enzyme using GMAC, similar to the conversion (Y%) of free enzyme. High free enzyme activity is expected, as it has greater mobility and access to the substrate [21]. The high conversion for both lipases can be attributed to their specificity for short- and medium-chain compounds, such as ethyl alcohol and lactic acid [78].

These results demonstrate that immobilization on functionalized activated carbon preserves the catalytic performance of lipases at levels close to those of the free enzyme while providing the robustness necessary to withstand the harsh conditions of esterification reactions. Moreover, this approach addresses major limitations associated with free enzymes, including rapid deactivation, limited operational stability, and the inability to be efficiently recovered and reused [79].

These results demonstrate the suitability of the developed materials as effective supports for lipase immobilization, using ethyl lactate synthesis as a model esterification reaction. Although ethyl lactate was selected for its industrial relevance and low toxicity, the high ester conversion obtained indicates that the immobilized biocatalysts exhibit adequate stability and catalytic performance under esterification conditions. Therefore, the favorable results observed for this model reaction suggest that the proposed immobilization strategy and the resulting materials may be extended to the synthesis of other structurally related esters, particularly those of interest to the food, cosmetic, and pharmaceutical industries. Ethyl lactate is widely used as a flavoring agent, contributing buttery notes to dairy products and wines [80]. Previous studies have reported ester conversions of 87.32% using *Aspergillus fumigatus* lipase [81], 83.3% using immobilized *Candida antarctica* lipase B (CALB) [82], and 88% using Novozym 435 [83], placing the present results within the range of established biocatalytic systems and reinforcing the broader applicability of the developed derivatives.

### Operational Stability

**Fig. 6:**
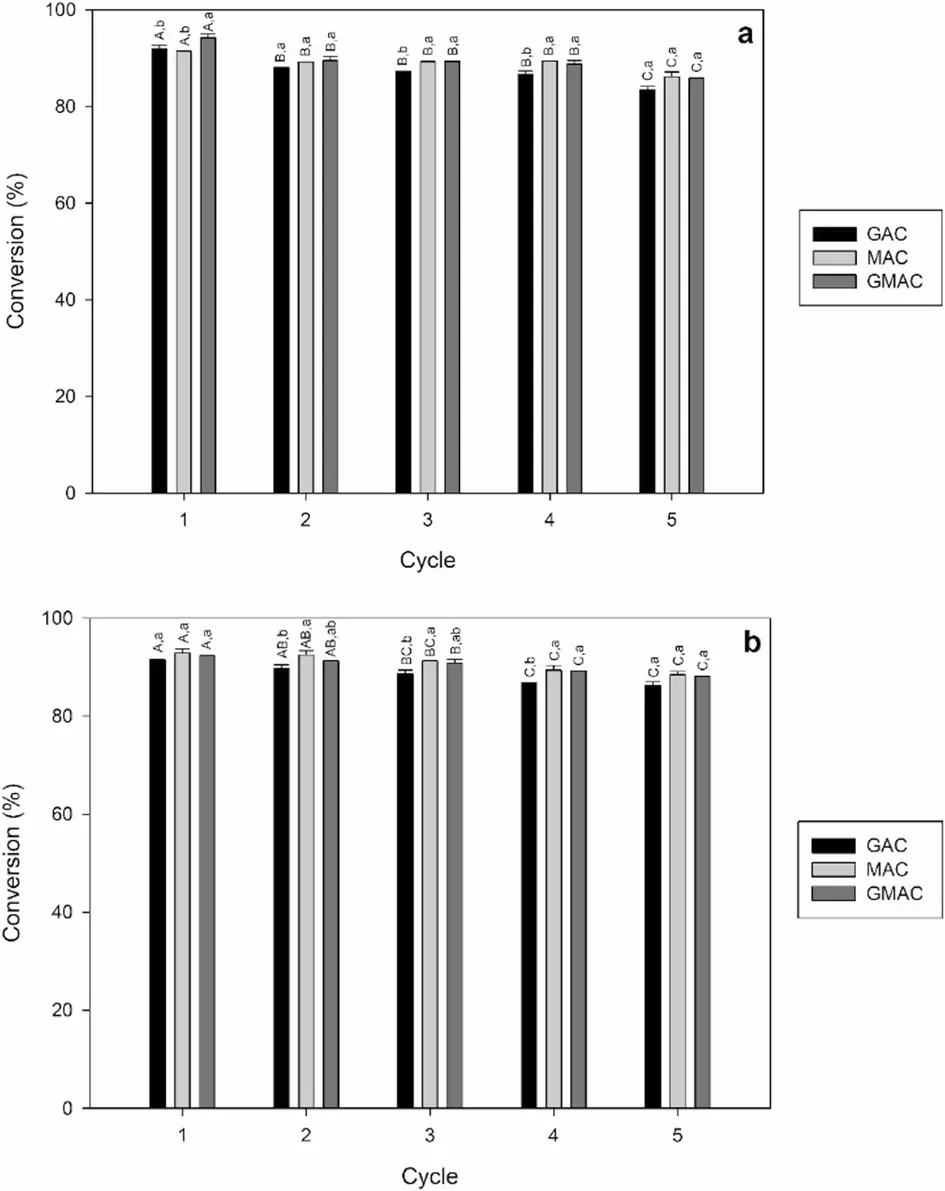
Conversion of ethyl lactate ester catalyzed by PPL (**a**) and CRL (**b**) immobilized on activated carbon functionalized with genipin (GAC), IDA + metal (MAC), and genipin + metal (GMAC) as a function of the number of use cycles (T = 40 °C, t = 4 h). *Means followed by the same lowercase letter in the sample and uppercase letter in the cycle of reuse for the same sample do not differ significantly from each other according to Tukey’s test (*P* < 0.05)

The main feature of the industrial application of immobilized enzymes is their operational stability, as reusing the biocatalyst reduces process costs [82]. To illustrate this point, Fig. 6 presents the cycles of use for lipase from porcine pancreas (a) and *Candida rugosa* (b) using the functionalized activated carbons based on Ethyl lactate conversion (%).

All derivatives exhibited stability during reuse for both enzymes, and the metalized supports had the highest conversion in the last cycle (MAC = 86.16 ± 0.947% and GMAC = 85.79 ± 0.117% for PPL; MAC = 88.46 ± 0.657% and GMAC = 88.14 ± 0.073% for CRL). Furthermore, compared to the unfunctionalized activated carbon, the functionalized-activated carbons showed greater stability (AC = 80.68 ± 0.888% in the last cycle) [24]. This may be due to strong interactions between the lipase structure and the functionalized supports, such as covalent bonds and electrostatic interactions, which mitigate biocatalyst losses via desorption [60]. Moreover, these results suggest that using genipin and iminodiacetic acid as crosslinking and chelating agents was an efficient method in preventing the leaching of metal ions from the carbon surface [84].

Both lipase sources showed successful results across the reuse cycles, with minimal activity loss throughout, although PPL was less stable in the final cycle (5th). A slight reduction in catalytic activity across cycles may be attributed to multiple deactivation mechanisms, including progressive structural denaturation of the enzyme due to prolonged exposure to organic solvents and esterification reagents, which may alter the conformation of the active site. In addition, successive cycles may promote residual enzyme leaching from fractions that were not covalently bound to the support. Another contributing factor may be the accumulation of by-products or water generated during the esterification reaction on the support surface, which can block pores and hinder substrate diffusion toward enzymes confined within the support’s internal structure, thereby reducing overall catalytic efficiency [82, 85, 86]. The highest ester conversion after 5 cycles (> 83%) suggests that the bonds between the enzyme and the functionalized supports increased the lipase’s structural rigidity and protected the biocatalyst from denaturation during successive uses [80]. Koutinas et al. [81] found 85% of ethyl lactate after 5 cycles using the commercial lipase Novozym 435. Furthermore, Brito et al. [82] state that washing the derivative with hexane after each cycle may have helped maintain its activity, as this non-polar solvent removes any product around the enzyme that may reduce its activity and limit substrate and product diffusion.

### Effect of Terc-butanol as a Solvent

The polarity of the esterification solvent was investigated by using terc-butanol as a solvent based on the conversion (Y%) in ethyl lactate. The study found Y(%) = 73.25 ± 0.62% for free PPL and Y(%) = 70.60 ± 2.97% for PPL-GMAC. On the other hand, it was reported that Y(%) = 77.49 ± 0.92 for free CRL and Y(%) = 75.17 ± 2.45 for CRL-GMAC. These findings are lower than those obtained using hexane as a solvent, but this is expected as lipase-mediated esterification occurs mainly in a hydrophobic medium (isooctane > petroleum ether > n-hexane > terc-butanol > terc-amyl alcohol). According to Subroto et al. [83], it is caused by a strong deformation due to the essential interactions between water and protein. Hydrophilic solvents influence the water content surrounding the lipase microenvironment at the active site, thereby affecting its conformation, structure, and activity. Further studies are needed to fully validate the method; however, promising conversion (Y > 70%) indicates that t-butanol could serve as a less toxic alternative to hexane in the synthesis of ethyl lactate using derivatives obtained in this study.

## Conclusion

The surface functionalization of activated carbon derived from sisal waste using genipin, iminodiacetic acid, metallic particles, and their combinations was successfully applied to immobilize lipases from porcine pancreas and *Candida rugosa* for the synthesis of ethyl lactate. Functionalization has proven to be a robust strategy, demonstrating that the proposed modifications ensure efficient binding and enhance catalytic activity through synergistic interactions between the modifying agents and the enzyme structure. All supports exhibited high immobilization efficiency, with the metalized activated carbon showing the highest hydrolytic activity, particularly the PPL immobilized on GMAC. Furthermore, this study makes an original contribution by valorizing sisal agro-industrial residues as support materials and employing genipin as a safer and more effective crosslinking alternative to glutaraldehyde. The use of genipin as a natural cross-linking agent has proven to be a superior alternative to conventional chemical reagents, providing greater process stability and safety while reinforcing the methodology’s sustainability. The promising high ester conversion rates after five reuse cycles indicate that the immobilization process effectively protects lipase from desorption and inhibition. The high operational stability suggests that immobilization protected the lipases from denaturation and desorption, establishing these biocatalysts as promising candidates for large-scale applications. The catalytic performance of these biocatalysts can also be extended to the synthesis of other esters, and, with further studies, they may be applied in continuous industrial systems.

## Data Availability

The data supporting this study’s findings are available from the corresponding author upon reasonable request.
